# Assessing the Fatty Acid, Carotenoid, and Tocopherol Compositions of Seeds from Apple Cultivars (*Malus domestica* Borkh.) Grown in Norway

**DOI:** 10.3390/foods10081956

**Published:** 2021-08-22

**Authors:** Milica Fotirić Akšić, Kristina Lazarević, Sandra Šegan, Maja Natić, Tomislav Tosti, Ivanka Ćirić, Mekjell Meland

**Affiliations:** 1 Faculty of Agriculture, University of Belgrade, 11080 Belgrade, Serbia; fotiric@agrif.bg.ac.rs; 2 Centre for Food Analysis, 11080 Belgrade, Serbia; kristina.lazarevic@cin.co.rs; 3 Institute of Chemistry, Technology, and Metallurgy, University of Belgrade, 11000 Belgrade, Serbia; sgaica@chem.bg.ac.rs; 4 Faculty of Chemistry, University of Belgrade, 11000 Belgrade, Serbia; mnatic@gmail.com (M.N.); tosti@chem.bg.ac.rs (T.T.); 5 Innovation Centre of Faculty of Chemistry Ltd., 11000 Belgrade, Serbia; ivankai@chem.bg.ac.rs; 6 Norwegian Institute of Bioeconomy Research, NIBIO Ullensvang, Ullensvangvegen 1003, N-5781 Lofthus, Norway

**Keywords:** seed oil, defatted seeds, linoleic acid, oleic acid, β-carotene, lycopene

## Abstract

Apple production generates large amounts of apple pomace including seeds, leading to high transportation costs, public health hazards and undesirable odor. A new reuse strategy of this kind of waste could solve environmental issues and/or create unconventional sources of health beneficial products. In total, seeds from 75 apple cultivars grown in Norway (both domestic and international) have been analyzed for the first time for oil content and fatty acid profile together with tocopherols and carotenoids quantification in defatted seeds. Seeds from cultivar Håkonseple had the highest oil content (22.10%), with linoleic, oleic acid, and palmitic acid as the most abundant fatty acids. The levels of β-carotene and lycopene carotenoids and α-tocopherol were the highest in defatted seeds of the cultivar Sureple Grøn. Principal component analysis separated cultivars according to the total oil content. The Norwegian apple cultivars Håkonseple, Kviteple, Tolleivseple, Vinterrosenstrips, and Tokheimseple are recommended for obtaining vegetable oil due to their high oil contents, while cultivar Sureple Grøn can be separated due to its high levels of β-carotene, lycopene and total tocopherols.

## 1. Introduction

Apple (*Malus* × *domestica* Borkh.) is economically and culturally the most important temperate fruit crop in the world. It ranks second among the most widely produced fruits in the world after banana. Due to climate adaptations and having a standing temperature between −30 °C and +40 °C, apple trees are grown in 96 countries that produce apples for their domestic markets and export [1]. Apple contributes significantly to human daily consumption due to having less perishability than most other fruits, year-round availability, good transportability, comparatively low price, and nutritional qualities [2]. The global acreage is ~4.7 mil ha, bearing ~87 mil tons of apples, where China produces ~42 mil tons, USA is in second place with ~5 mil tons and Turkey in the third place with ~3.6 mil tons [3].

Because of its precious characteristics (aroma, taste and its beneficial effects in counteracting obesity, cancer, cardiovascular disease, asthma, diabetes and others), out of the total production, about 70% of the fruit is consumed as fresh, while 25–30% of the fruit is processed [4,5]. Among numerous products, juice, concentrate, marmalade, jam, dried fruits and cider could be underlined as the most important. Based on Statista (2021) [6] and AICV (2020) [7], more than 2 million liters of apple juice and more than 1.5 million liters of cider, respectively, were produced in the EU in 2017. The total industrial production process is about 70–75% juice with 25–30% apple pomace and 5–11% sludge. Apple pomace consists mainly of apple skin/flesh (95%), seeds (2–4%) and stems (1%) [8]. According to Bhushan et al. [9], several million tons of apple pomace and several hundred thousand tons of apple seeds are generated worldwide.

The bulky nature of apple pomace and its susceptibility to microbial decomposition are leading to public health hazards. Even dumping of such material is a big problem due to the high transportation costs and undesirable odor [9]. In the past, apple pomace was used as animal feed, fuel for boilers, added to soil as a conditioner, and as a substrate for microbial growth and later in the production of value-added products such as organic acids, enzymes, single cell proteins, low alcoholic drinks, ethanol, biogas, pigments and baker’s yeast [10,11,12]. Recent studies have reported that the side-products of the *Malus* genus can be used in the pharmaceutical and natural cosmetic industries for the production of perfumes, toiletries and chemical additives [13,14]. Still, there is no systematic collection and utilization of this material; thus, a valuable product with a large industrial potential remains unexploited [15].

Apple seeds have been shown to be rich in bioactive compounds such as proteins, carbohydrates, minerals, unsaturated (monounsaturated and polyunsaturated) fatty acids (mostly oleic acid and linoleic acid), tocopherols and tocotrienol homologues (tocochromanols), carotenoids, dietary fiber and polyphenolics [16]. Apple seeds contain high amounts of fat that can reach up to 29% of oil but do not contribute to cholesterol formation in humans [17]. Thus, this kind of oil can be considered as a high-quality edible vegetable oil [18,19,20,21] proved to have antioxidant, antimicrobial and antiproliferative properties. Oil obtained from apple seeds is light yellow and aromatic. Nevertheless, seeds also store amygdalin (cyanogenic glycoside), which is potentially toxic in the presence of enzymes resulting in the releasing of hydrogen cyanide. The extraction of amygdalin can be carried out using a Soxhlet extractor, an ultrasonic bath, or the solid-phase extraction (SPE) method. Polar solvents are suitable for the extraction of amygdalin. Mostly these are ethanol, methanol, ethyl acetate, and water, but also 0.1% citric acid solution under reflux is used to increase the efficiency of extraction. To obtain a high-quality oil free of amygdalin, the extraction procedure should be optimized or cold-pressing can be carried out [22].

After oil extraction (up to 29% of seed), the press cake is discarded as waste. Such waste materials could present a reservoir of low-cost natural compounds needed for human nutrition (carbohydrates, carotenoids, tocopherols, proteins, polyphenols, pectin, minerals, and acids), especially value-added allergy-free (such as gluten and lactose free) products [23]. Tocopherols (vitamin E), lipid-soluble tocochromanols synthesized by photosynthetic organisms, are one of the most important natural antioxidants [24]. The role of four homologues (α, β, γ and δ) is to protect polyunsaturated fatty acids against oxidation and support chemical processes through cell membranes, especially in seed storage and during low temperatures [25]. This level is increasing due to stressful environmental conditions. Tocopherols have a high impact on human health by protecting the neurological system, preventing heart disease and prostate cancer, and having anti-inflammatory and antidiabetic effects [26]. Carotenoids, lipophilic compounds that are commonly found in photosynthetic tissues, are responsible for the red, orange and yellow nuances of fruits, and are having an important role in attracting pollinators, seed dispersal, and phytohormone production. Carotenoids play important roles during the initial phases of seed imbibition, germination and seedling development. Consumption of them is connected with the inhibition of carcinogenesis, cardiovascular and other degenerative diseases and enhancement of the immune response and cell defense [27].

Fruit production in Norway is located in the southeastern part (around lakes) and in the southwestern part (around fjords), which are the most northerly fruit tree-producing areas in the world. Apples are the largest fruit crop produced in Norway. Consumption of fruit is increasing and there is a high demand for locally, sustainably produced apples of high quality. The acreage of Norwegian commercial apple production in 2020 was 1538 ha, where a total of 6783 tons were freshly consumed and 5674 tons were used for juice processing [28]. The by-product of the juice industry—apple pomace—is a waste in Norway, currently has no commercial value, and is used for feeding animals.

Recently, an interest in the usage of agro-industrial waste and unconventional oil sources has gained more attention. This kind of by-product from the apple industry can lower disposable problems, and obtained products can be a potential source of natural bioactive compounds with high biological importance and economically feasible production. The aim of this study was to analyze the seed oil from different traditional Norwegian apple cultivars, for the first time, and recommend the cultivars that can be used for oil production. Regarding the fact that the majority of scientists have dealt with tocopherol and carotenoid composition in seed oils [14,18,29], but not many have discussed tocopherols and carotenoids in residues after oil extraction, another goal was to quantify their presence in defatted seeds.

## 2. Materials and Methods

### 2.1. Plant Material

In this study, fruits from a total of 75 apple accessions maintained in the ex situ collection located in western Norway (Hjeltnes college, a municipality of the Hardanger district—lat 60°33′ N, long 6°55′ E) were sampled during the falls of 2017 and 2018 (Table 1). Most sampled accessions are considered traditional Norwegian cultivars. However, some cultivars represent foreign cultivars with a longer or shorter tradition of commercial production in Norway. Each accession was represented with five trees. Cultivars were grafted on the M 26 rootstock and planted in the period 1995–2000. Planting distance was 3 × 5 m with an East–West row orientation and trained as spindle trees. Orchard floor management consisted of grass in the interrow and a 1 m-wide herbicide strip in the intrarow space. The trees were not irrigated. All trees received the same amount of fertilizers each spring based on soil analysis. The trees were managed according to the general agricultural practices for the area including the plant protection program. Harvest time was according to the commercial standards for maturity based on fruit color, seed development and firmness.

Fruits from each cultivar were picked during full maturity (when fruits had typical color and taste and when seeds became brown). Seeds were extracted from 20 fruits (picked from five different trees and from all around the canopies), washed up with tap water and air dried for ten days. After, seeds were bagged and stored in a cool place until analysis.

### 2.2. Standards and Chemicals

Lutein, zeaxanthin, β-cryptoxanthin, canthaxanthin, astaxanthin, apocarotenal, physalien, β-carotene, lycopene and tocopherols (α, β, γ and δ) were from Sigma-Aldrich, Inc. (St. Louis, MO, USA). Acetonitrile, methanol, ethanol, dichloromethane, acetone, n-hexane and ascorbic acid were of analytical-grade purity and purchased from Merck (Darmstadt, Germany). Water was prepared using a Millipore Simplicity 185 S.A., 67120, water purification system (Molshem, France). The standard FAME mixture of 37 components was purchased from Food Industry FAME Mix, RESTEK (Bellefonte, PA, USA) (lot 24676).

### 2.3. Oil Extraction and Fatty Acid Methyl Esters Determination

The oil extraction and determination of fatty acid methyl esters of apple seeds were carried out according to standard method ISO 12966-2:2017 [30] and ISO 12966-4:2015 [31]. Seed oil extraction was performed by Soxhlet extractor (Soxtherm-Gerhard) using n-hexane as a solvent. Oil was extracted with 130 mL of n-hexane from 10 g of seed powder using a Soxhlet extraction system for 3 h. At the end of the extraction, the solvent was evaporated. The obtained oils were flushed with nitrogen to remove the residual traces of n-hexane and stored in the dark at 4 °C. Fatty acid methyl esters (FAME) were prepared using transmethylation under alkaline conditions, following EN ISO 12966-2:2017 [30]. In a 10 mL screw-top test tube, approximately 0.1 g of the extracted oil was weighed and dissolved in 2 mL n-hexane. After the addition of 1 mL of 2 mol/L methanolic potassium hydroxide solution, the tube was vortexed for 2 min at room temperature, and centrifuged at 4000 rpm for 5 min. After 2 min, 2 mL of sodium chloride solution (40 g of sodium chloride in 100 mL of water) was added and the tube was shaken briefly. The solution was neutralized by adding 1 g of sodium hydrogen sulfate, anhydrous. After the salt had settled, 1 mL of the upper phase was transferred to a 2 mL vial for FAME analysis.

**Figure 1:**
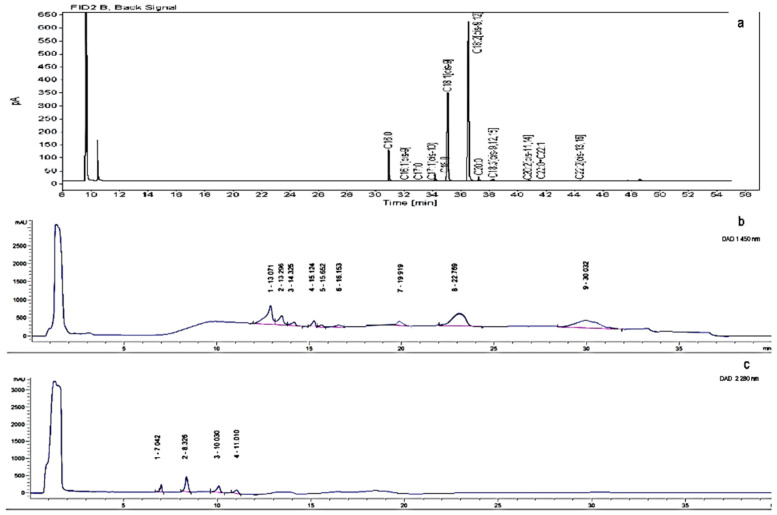
The representative chromatograms of (**a**) fatty acids, (**b**) carotenoids (numbers respond to compounds in Table 2), and (**c**) tocopherols (numbers respond to compounds in Table 3).

Fatty acid methyl esters were analyzed by gas chromatography, according to [31] EN ISO 12966-4:2015, using a GC-FID Agilent 7890B GC System with flame ionization detection (FID). A fused-silica capillary column type CP-Sil 88 for FAME 100 m × 0.25 mm df = 0.2 µm was used. The flow rate of carrier gas was 1.0 mL/min. Injector and detector temperatures were 250 °C and 270 °C, respectively. The oven temperature was programmed to start with a temperature of 80 °C, then to rise to 220 °C at a rate of 4 °C/min and to maintain that temperature for 5 min, then to rise to 240 °C at the rate of 4 °C/min, and to maintain that temperature for more 10 min. The sample injection volume was 1 μL. Total run time for one cycle was 55 min. Fatty acid identifications were based on retention times by comparing with those of the standard FAME mixture containing 37 components. All other reagents were of analytical grade. Quantification of individual fatty acids was based on the peak area obtained, without any corrections. Fatty acid analysis was performed in duplicate for single samples, and average values were reported. The average relative standard deviation (RSD) of repeatability for minor components (components present at less than 1%) was 5%, while the average RSD for the components present in percentages greater than 1% was 2–3%. Limit of quantification was 0.01%. The results are given in Supplementary Tables S1 and S2. The representative chromatogram of fatty acids is given in Figure 1a.

### 2.4. Carotenoids and Tocopherol Analysis

#### 2.4.1. Extraction Procedure

Extraction was performed by ultrasonication of 5 g of defatted seeds of each apple variety separately with solvents of different polarity (hexane, acetone, and ethanol) and the mixtures were left in an ice-cold VF ultrasonic bath (frequency 40 kHz, volume 4 L) for 15 min under a stream of nitrogen. In order to improve the efficacy of total carotenoids extraction, solvents of different polarity were used. Hexane and acetone were used for extraction of less polar carotenoids (lycopene, β-carotene, β-cryptoxanthin, canthaxanthin), while ethanol was used for extraction of other more polar carotenoids (lutein, zeaxanthin, apocarotenal, physalien). The mixtures were centrifuged at 8000× *g* for 10 min and supernatants, which contained extracted carotenoids and tocopherols, were removed. The solid residues were re-extracted with fresh extraction solvent, applying the same procedure. The obtained extracts for each solvent were filtered, combined, and evaporated under N_2_. The residues were re-dissolved in 1 mL mixture of dichloromethane/acetone/ethanol (6:2:2, *v*/*v*/*v*), whereby the ethanol contained 0.5 g of ascorbic acid per 100 mL. This solvents mixture enables simultaneous extraction of less and more polar carotenoids [32]. The obtained extracts were filtered through 0.22 μm and a volume of 100 µL was injected into the HPLC column.

#### 2.4.2. HPLC Analysis

An Agilent 1200 HPLC system (Agilent, Santa Clara, CA, USA) equipped with a Quat Pump (G1311B), Injector (G1329B) 1260 ALS, TCC 1260 (G1316A), Detector 1260 DAD VL+ (G1315C), and thermostated column compartment was used. Chromatographic separation was performed on octadecyl silica as a stationary phase (Nucleosil C18 analytical column, 4 mm × 150 mm, 5 μm) using a mobile phase consisting of deionized water (A), acetonitrile (B), and methanol (C) [33]. The optimized HPLC method was developed to allow appropriate separation of individual carotenoids and tocopherols. The gradient protocol was: 0–4 min 15% A, 60% B, 25% C, 4–6 min 15% A → 0% A, 60% B → 70% B, 6–34 min 0% A, 70% B, and 30% C, 34–35 min 0% A → 15% A, 70% B → 60% B, 30% C → 25% C, 35–40 min 15% A, 60% B, 25% C. The mobile phase temperature was set at 30 °C, the flow rate was 1.0 mL/min. UV–vis diode array detection was set at 450 nm for carotenoids and at 280 nm for tocopherols. Carotenoids and tocopherols were identified by comparing their retention times and spectral data with those of the authentic standards. Quantification was performed by comparing peak area with standard reference curves.

#### 2.4.3. Method Validation for Carotenoids and Tocopherols

The carotenoid standards lutein, zeaxanthin, β-cryptoxanthin, and physalien were prepared at a concentration of 1.00 mg/mL in acetone-ethanol (1:1). The beta carotene and lycopene were prepared at a concentration of 1.00 mg/mL in acetone [34] and dichloromethane, respectively. The standard solutions of tocopherols were prepared in an extraction mixture (dichloromethane/acetone/ethanol (6:2:2, *v*/*v*/*v* with ascorbic acid)) and a volume of 20 μL was injected. The parameters of method validation for carotenoids are given in Table 2 and the parameters of method validation for tocopherols are given in Table 3. The representative chromatograms of carotenoids and tocopherols are given in Figure 1b,c, respectively.

### 2.5. Statistical Analysis

Basic statistics: minimum (min), maximum (max), median and standard deviation (SD) of the obtained amounts of fatty acids, carotenoids and tocopherols in apple cultivar seeds were calculated with Excel software ((Microsoft Office Professional Plus 2013, Santa Rosa, CA, USA).

Principal component analysis (PCA) is one of the most widely used multivariate methods that reduces the dimensionality of the original dataset to a new set of non-correlated variables, called principal components (PCs), without losing much information. The results of a PCA are usually interpreted in terms of components (the transformed variable values corresponding to a data point), scores which correspond to the original samples, and loadings which correspond to the original variables. Graphical presentation of PCs as 2D or 3D patterns gives an overview of the relations between variables and between samples looking for groups and trends, sorting out outliers [35,36]. Principal component analysis (PCA) was performed with the PLS Toolbox statistical package (version 5.7, Eigenvectors Inc.) from MATLAB version 7.4.0.287 (R2007a) (MathWorks INC, Natick, MA, USA) on a data matrix containing amounts of carotenoids, tocopherols and fatty acids in apple cultivar seeds.

The data overview is obtained by using a singular value decomposition algorithm (SVD) and a 0.95 confidence level for Q and T2 Hotelling limits for outliers. In order to obtain variables on the same scale, the data pretreatment method called autoscaling (centering and rescaling to unit standard deviation) was performed.

## 3. Results

### 3.1. Oil and Fatty Acids Determination

Results showed a high variability in total oil content among the apple seeds from the different cultivars (Table S1). The oil content ranged from 7.73 up to 22.10%. The average oil content in investigated apple seeds was 16.00 ± 3.39 g/100 g seeds. The lowest oil content was determined in seeds from the cultivar Bramley Seedling (7.73%), and it was also found in low amounts (less than 10 g/100 g) in the cultivar Haugeeple (9.69%), Knuteple (9.96%), Leiknes (9.71%), Oster (8.50%), Raud Gravenstein (9.69%), and Rossvolleple (8.67%). The sample with the highest oil content was sample Håkonseple, with oil content of 22.10%. Oil content higher than 20% was also determined in the cultivars Kviteple, Rubinstep, Tolleivseple, Vinterrosenstrips, and Tokheimseple, and the range was from 21.21% to 20.22%.

In total, sixteen fatty acids were identified and quantified, where the most abundant were linoleic acid, oleic acid and palmitic acid, in decreasing order (Table S1). These three unsaturated fatty acids represented more than 95% of the total fatty acid contents. Of the total fatty acids content, linoleic acid averaged 59%, oleic acid averaged 29%, and palmitic acid 7%. Saturated fatty acids, stearic and arachidic acid, were represented in a smaller but significant amount. Linoleic acid was the most abundant fatty acid, and its content in all samples was presented greater than 50%, with range from 50% to 66%. Cultivar Bramley Seedling had the highest amounts of linoleic acid (66.73%), omega-6 fatty acids (66.98%) and polyunsaturated fatty acids (67.87%) (Tables S1 and S2). Palmitoleic acid, heptadecanoic acid, cis-10-heptadecenoic acid, α-linolenic acid, 11-eicosenoic acid, eicosadienoic acid, arachidonic acid, heneicosanoic acid, behenic acid, lignoceric acid and docosahexaenoic acid were present in all samples in very small amounts (<0.5%).

### 3.2. Determination of Carotenoids

**Figure 2:**
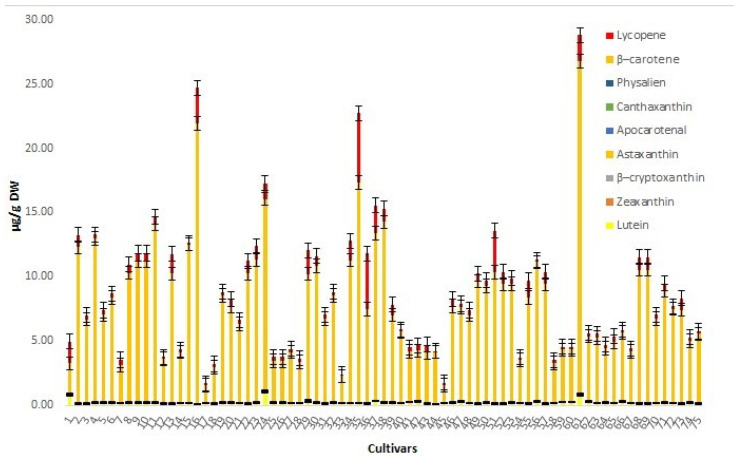
The amounts and standard errors of carotenoids in analyzed seeds of apple cultivars grown in Norway.

The carotenoids lutein, zeaxanthin, β–cryptoxanthin, astaxanthin, canthaxanthin, and physalien belong to the group of xanthophyll. The presence of alcohol, ketone, aldehyde, acid, or epoxide groups in their structure makes them less lipophilic. On the other hand, apocarotenal, β–carotene and lycopene belong to a group of carotenoids which contain only a hydrocarbon chain without any functional group; therefore, they are very lipophilic [37]. The extraction procedure was adapted to the different polarity of these two groups. Ethanol and acetone were applied for more polar carotenoids, while hexane, which is a nonpolar solvent, was used for the extraction of nonpolar carotenoids [32]. The amounts of each carotenoid content expressed as micrograms per gram of dry weight (μg/g DW) are presented in Figure 2 and the statistical parameters (min, max, median, SD) are given in Table 4.

The dominant carotenoids in the carotenoid profile of investigated seeds from apple cultivars are β-carotene and lycopene in a range of 1.370–25.800 μg/g DW and 0.080–5.370 μg/g DW, respectively. The cultivars with the highest content of these pigments in seeds were Sureple Grøn (25.800 and 2.034 μg/g DW, respectively), Furuholm (21.800 and 2.754 μg/g DW, respectively), and Laxton Exquisite (17.120 and 5.370 μg/g DW, respectively) while the lowest amounts were in the cultivars Garborg (1.415 and 0.1176 μg/g DW, respectively) and Quinte (1.375 and 0.1677 μg/g DW, respectively). At the same time, the cultivars Garborg and Quinte had the lowest determined amount of total carotenoids. Most other carotenoids (lutein, zeaxanthin, cryptoxanthin, astaxanthin, apocarotenal, and physalien) were present in much lower amounts. Among them, a lutein in slightly higher amounts was found in seeds of cultivars Herrasaleple (0.936 μg/g DW), Sureple Grøn: (0.698 μg/g DW), and Aroma (0.694 μg/g DW).

### 3.3. Determination of Tocopherols

**Figure 3:**
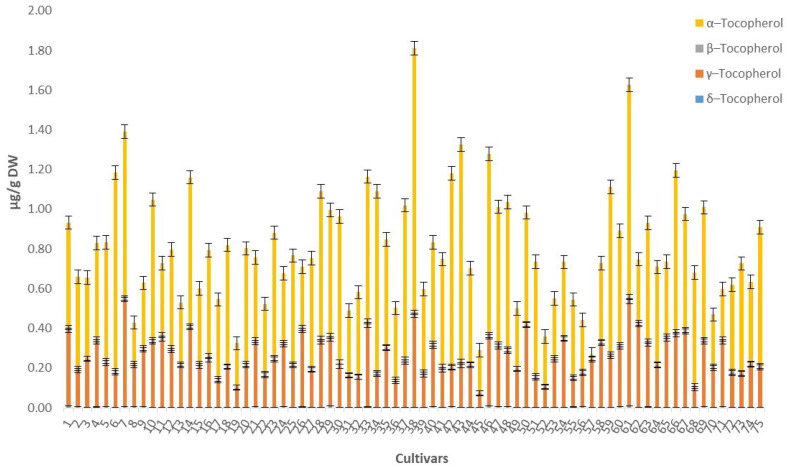
The amounts and standard errors of tocopherols in investigated seeds of apple cultivars grown in Norway.

The amounts of each tocopherol content in cultivar seeds expressed as micrograms per gram of dry weight (μg/g DW) are given in Figure 3 and the statistical parameters (min, max, median, SD) are given in Table 5. In investigated residues after oil extraction, the dominant tocopherol isomers were α- and γ-tocopherols (mean values 0.539 and 0.258 μg/g DW, respectively) while β- and δ-tocopherols were detected in lower amounts (mean values 0.009 and 0.003 μg/g DW, respectively).

The largest amounts of γ-tocopherol, above 1 μg/g DW, were detected in the cultivars Løeeple (1.331 μg/g DW), Paradiseple (1.093 μg/g DW) and Sureple Grøn (1.067 μg/g DW), while γ-tocopherol concentration was the highest in the cultivars Charlamowsky (0.540 μg/g DW), Sureple Grøn (0.527 μg/g DW) and Løeeple (0.462 μg/g DW). At the same time, the largest amounts of total tocopherols were detected in the cultivars Løeeple, Sureple Grøn and Charlamowsky, 1.811, 1.626 and 1.391 μg/g DW of the residue, respectively. This variation in tocopherol content and composition was attributed to genetic factors.

### 3.4. Principal Component Analysis (PCA)

**Figure 4:**
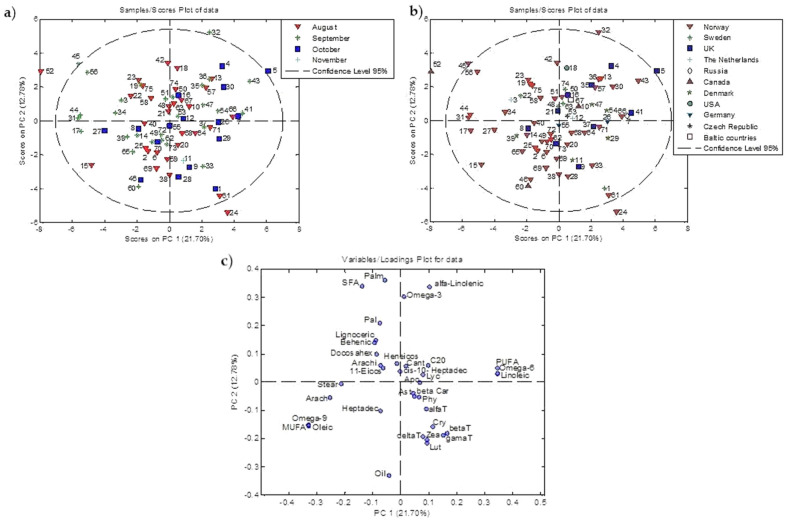
PC1−PC2 Score plots. (**a**) The classes are marked based on ripening time; (**b**) The classes are marked based on country of origin; (**c**) Loading plot. Please see the Appendix A for the list of abbreviations.

PCA resulted in a four-component model, which explains 49.72% of total variance (PC1 21.70%, PC2 12.78%, PC3 8.35%, and PC4 6.88%). The cultivars Bramley Seedling, Herrasaleple, Knuteple, and Quinte are outside the T2 Hotelling limits. In the scores plot of data, cultivar Bramley Seedling had the highest positive value of PC1, and the loadings plot indicates that the highest values of amounts (max of range) of linoleic acid (66.73%), omega-6 fatty acids (66.98%) and polyunsaturated fatty acids (63.87%) are discriminative factors, which influence the position of cultivar Bramley Seedling outside of T2 Hotelling limits. Cultivar Herrasaleple is recognized as the outlier due to having the highest amounts of carotenoids: lutein and zeaxanthin (0.936 and 0.109 μg/g DW, respectively). In cultivar Knuteple, the highest amount of omega-3 fatty acids (1.26%) was detected. Cultivar Quinte was characterized with the highest value (13.75%) of saturated fatty acids (SFA). In Figure 4, scores and loading plots of PC1 and PC2 are given.

## 4. Discussion

### 4.1. Oil and Fatty Acids

The results are at the same level as published data regarding the fatty acid profiles and the content of triacylglycerols. Large variations in the total oil contents among studied cultivars are reported to be genotype dependent. Górnaś et al. [13] studied crab and dessert apple seeds and reported seed oil yield in a range from 12.06 to 27.49% dry weight, and it was significantly dependent on the cultivar. In another study, Matthäus and Özcan [38] obtained 21.9% and 25.6% of seed oil content of the two apple cultivars Golden Delicious and Starking, respectively, while Lei-Tian et al. [20] reported apple seed oil with a range from 20.6% to 24.3%.

The most dominant fatty acids in the investigated apple seeds were linoleic acid (59.37–67.94%), oleic acid (20.68–29.00%), and palmitic acid (5.78–8.33%). Arain et al. [18] found oil content in the seeds of cultivars Royal Gala, Red Delicious and Golden Delicious from 26.8% up to 28.9%. In addition, Bada et al. [39] found the content of oil ranged from 19.67 to 22.73% of seeds from seven apple species from Asturias (Spain) and linoleic acid was reported as the main component in Limón Montés (60.78%) and Riega (60.01%). Linoleic acid was dominant in Royal Gala (45.1%), Red Delicious (47.8%), and Golden Delicious (40.5%) [13]. In other studies [29,40], the Fuji and New Red Star seed oils mainly consisted of linoleic acid (50.7–51.4 g/100 g) and oleic acid (37.49–38.55 g/100 g). These authors investigated dessert and cider apples (*Malus domestica* Borkh.) cultivars of different origin. Quantitatively, the oils were rich in linoleic acid (32.5 to 49.7 g/100 g) and oleic acid (15.1–33.3 g/100 g), while the contents of saturated fatty acids were up to ten times lower. The potential benefit of increasing dietary intake of linoleic acid is connected to cardiovascular and mental health, and anti-cancerogenic and anti-diabetic effects [40]. Oleic fatty acid consumption has been related to improved pancreas and liver secretory activity, protecting the cells from inflammation and fighting against coronary heart disease, rheumatoid arthritis, and cancer [41]. Linoleic, oleic and palmitic acid, the rest of the fatty acids determined in this study, had minor quantities, which supports the findings of Arain et al. [18] who also identified linolenic, palmitoleic, heptadecanoic, and 11-ecosenoic fatty acids in trace amounts (<1%).

Moreover, apple cultivars showed pronounced differences in yields, numbers, and weights of their seeds [29] and this was also the case for the investigated samples presented herein.

### 4.2. Carotenoids

The examination of the carotenoids in plants is mainly focused on the photosynthetic tissues and fruits. In seeds, they limit the levels of free radicals and reduce peroxidase activity, thus reducing seed ageing and loss of seed viability [42]. It has been noticed that during germination, the carotenoid content first increases slightly, and after the end of germination, it continues to grow sharply, even ten times more than the initial carotenoid content. This process is accompanied by a change in the presence of carotenoids, which reduces the presence of xanthophylls. There are few studies which compare the carotenoid contents of wild and domesticated plant species. Recent research has shown that wild legume seeds have higher levels of total carotenoids compared to their domesticated relatives. This is attributed to a side effect of selection for other desired traits, such as seed propagation mechanisms, seed storage and taste selections [43].

In the investigated press cake from apple seeds grown in Norway, the highest level of carotenoids were β-carotene and lycopene. β-carotene is a thermolabile orange pigment, and oxygen sensitive, and in most cases, it is connected with heart disease (it lowers LDL-cholesterol) and cancer. It is also a precursor of vitamin A, which is necessary for vision and cell proliferation. On the other hand, diets rich in lycopene are associated with lower risk of cancer and all kind of degenerative diseases [44,45].

Unlike seeds, some authors found higher amounts of carotenoids in the flesh and especially in the peel of cultivars with different external coloration (green, yellow and red) [46]. Also, Fromm et al. [47] reported carotenoid concentration from 0.10 to 1.58 mg/100 g oil in seed oils recovered from six cultivars of apples. Amariz et al. [48] determined 3.9 μg/g of total carotenoids in apple seeds.

### 4.3. Tocopherols

In previously investigated apple seed oils, the most abundant isomer was β-tocopherol; therefore, it is expected that the residue after oil extraction will not be rich in this tocopherol [14,47,49].

In the pressed cake of the studied apple cultivars that are grown in Norway, α-tocopherols were the most abundant, followed by γ-tocopherols. Although it is well known that α-tocopherol is the most abundant in leaves, whereas γ-tocopherol is dominant in seeds, some species such as sunflower, olive, safflower or grape store α-tocopherol as the main tocopherol form in seeds [24].

According to Górnaś et al. [14], seeds from the apple cultivars ‘Antej’ and ‘Beforest’ were analyzed and α-tocopherol ranged from 17.22 to 25.79 mg/100 g dry weight basis (dwb), β-tocopherol between 7.53 and 29.05 mg/100 g dwb, γ-tocopherol up to 13.82 mg/100 g dwb and δ-tocopherol within the range 0.16–10.79 mg/100 g dwb. The α:β:γ:δ tocopherol ratio in those two cultivars was 1.7:1.5:1.3:1.0 and 2.1:2.0:1.3:1.0, respectively.

All isomers (α-, β-, γ- and δ-) of tocopherol belong to the vitamin E active compounds. Their importance is reflected in the protection of polyunsaturated fatty acids against peroxidation [37]. They have various positive effects on human health, especially against cancer, heart disease, and other chronic ailments. Unfortunately, according to Fernández-Marín et al. [50], a decrease in concentration of carotenoids in legume seeds has been observed because of domestication, where modern cultivars are showing much lower levels of carotenoids compared to wild relatives, so such a trend should be investigated in apple wild genotypes and cultivars too.

### 4.4. Principal Component Analysis

Based on ripening time and based on country of origin, some conclusions could be drawn. According to the graph of loadings (Figure 4c), going along the PC1 axis, in the upper left part of the scores graph, cultivars are grouped according to their total amount of saturated fatty acids, as well as by individual content of saturated (palmitic, lignoceric, behenic, arachidonic, 11-eicosenoic, heneicosanoic) and some unsaturated omega 3 fatty acids (palmitoleic and docosahexaenoic) in their seeds. These cultivars were ripened in August and September. In the lower left part of the scores graph cultivars are grouped with similar amounts of saturated fatty acids (stearic, arachidic, and heptadecanoic), total amounts of monounsaturated fatty acids, omega 9, and with a similar amount of oleic acid, which is monounsaturated. These cultivars were ripened from August to October. In the upper right part of the scores graph, cultivars are grouped with similar amounts of omega 3 and α-linolenic, as with the total amount of PUFA, omega 6 fatty acids, and linoleic acid. These cultivars were ripened from August to October. The cultivars that have similar amounts of carotenoids and tocopherols in the seeds and were ripened from August to November are positioned in the lower right part of the scores graph.

Generally, cultivars with negative values of PC1 and PC2 are similar in content of saturated, monounsaturated fatty acids, carotenoids and tocopherols and belong mostly to the group of Norway cultivars. Cultivars with positive values of PC1 and PC2, which have similar content of polyunsaturated fatty acids, have different geographical origins.

## 5. Conclusions

Seeds obtained from apple cultivars grown in Norway (domestic and international) were investigated for the first time, in order to determine fatty acids, carotenoids and tocopherols. Taking into account geographical origin, in general, Norwegian cultivars are distinguished by a similar content of saturated, monounsaturated fatty acids, carotenoids and tocopherols. In addition, cultivars with similar content of saturated and monounsaturated fatty acids ripen mainly in August and September. On the other hand, varieties that have a similar content of polyunsaturated fatty acids are of different geographical origin and ripening time. Generally, seeds from the investigated apple cultivars could be separated into three different groups: one with similar amounts of omega 3 and α-linolenic acids, the second having similar total amounts of polyunsaturated fatty acids, omega 6 fatty acids, and linoleic acid, and the third group of seeds being characterized by similar amounts of carotenoids and tocopherols. The fatty acids profile could not provide a clear separation between the cultivars, but total oil content could distinguish between the cultivars. Therefore, seeds from several Norwegian apple cultivars (Håkonseple, Kviteple, Tolleivseple, Vinterrosenstrips, and Tokheimseple) could be further recommended for processing. Seeds from cultivar Sureple Grøn can be underlined due to its high levels of β–carotene, lycopene and total tocopherols.

Altogether, our findings could be considered valuable when a strategy to re-evaluate waste is created. This is the first study regarding effective reuse of by-products from the apple industry by producing oil and a defatted oil cake rich in tocopherols and carotenoids. Based on oil content analysis and favorable fatty acid composition, Norwegian apple seeds were shown to be a good source of fatty acids. Its oil has the potential to be used as an edible oil, which is valuable in the search for unconventional sources of health beneficial products. In addition, having in mind the fact that defatted oil cakes received little attention in general, studying other plant seeds is a new way to identify new sources important for good nutrition.

## Figures and Tables

**Table 1:** Investigated apple cultivars, country of origin, and ripening time.

No.	Cultivar	Origin	Ripening Time	No.	Cultivar	Origin	Ripening Time
**1**	Aroma	Sweden	October	**39**	Magelemer	Denmark	Sepember
**2**	Bananeple	Norway	September	**40**	Marta-Moster	Norway	Sepember
**3**	Beauty of Bath	UK	September	**41**	Norfolk Royal	UK	Sepember
**4**	Belle de Boskoop	The Netherlands	October	**42**	Oster	Norway	September
**5**	Bramley Seedling	UK	October	**43**	Paradiseple	Norway	August
**6**	Brureple	Norway	September	**44**	Prins	Norway	August
**7**	Charlamowsky	Russia	September	**45**	Quinte	Canada	August
**8**	Cox’s Orange	UK	October	**46**	Raud Granat	Norway	October
**9**	Cox’s Pomona	UK	October	**47**	Raud Gravenstein	Denmark	September
**10**	Early Red Bird	Canada	August	**48**	Raud Säfstaholm	Sweden	August
**11**	Elraud Pigeon	Denmark	November	**49**	Raud Sommerkavil	Norway	August
**12**	Elstar Boerekamp	The Netherlands	October	**50**	Ribston	UK	October
**13**	Franskar	Norway	August	**51**	Rondestveit	Norway	August
**14**	Fristeren	Norway	August	**52**	Rossvolleple	Norway	August
**15**	Fuhr	Norway	September	**53**	Rubinstep	Czech Republic	October
**16**	Furuholm	Norway	August	**54**	Signe Tillish	Denmark	September
**17**	Garborg	Norway	August	**55**	Silkepele	Germany	Sepember
**18**	Geneva Early	USA	August	**56**	Sitroneple	Norway	August
**19**	Grønt Laupsaeple	Norway	August	**57**	Stor Granat	Norway	Sepember
**20**	Gul Granat	Norway	September	**58**	Storesteinseple	Norway	Sepember
**21**	Gullspir	UK	August	**59**	Strutar	Norway	Sepember
**22**	Gyldenkoks Astrakan	Sweden	August	**60**	Summered	Canada	September
**23**	Haugeeple	Norway	September	**61**	Sureple Grøn	Norway	Sepember
**24**	Herrasaleple	Norway	September	**62**	Sysekavil	Norway	August
**25**	Hjartneseple	Norway	September	**63**	Sävstaholm	Sweden	August
**26**	Husmoreple	Germany	October	**64**	Tolleivseple	Norway	Sepember
**27**	Høyneseple	Norway	October	**65**	Tokheimseple	Norway	August
**28**	Håkonseple	Norway	October	**66**	Tormodseple	Norway	Sepember
**29**	Ingrid Marie	Denmark	October	**67**	Transparente Blanche	Baltic countries	August
**30**	Kaupanger	Norway	October	**68**	Tveiteple	Norway	September
**31**	Kavill	Norway	August	**69**	Ulgenes	Norway	Sepember
**32**	Knuteple	Norway	August	**70**	Vanleg Torstein	Norway	October
**33**	Kviteple	Norway	August	**71**	Vintergul	Norway	Sepember
**34**	Langballeeple	Norway	August	**72**	Vinterrosenstrips	Norway	August
**35**	Laxton Exquisite	UK	September	**73**	Worchester Pearmain	UK	September
**36**	Leiknes	Norge	September	**74**	Ølands Kungseple	Sweden	August
**37**	Lord Lambourne	UK	September	**75**	Øskhaug	Norway	September
**38**	Løeeple	Norway	September				

**Table 2:** Parameters of method validation for carotenoids ^1^.

No.	Compound	RT (min)	Regression Equation	*R* ^2^	LOD	LOQ	Recovery (%)	CV (%)
1	Lutein	13.071	y = 1.235x − 0.074	0.994	0.054	0.178	94.2–103.8	3.45
2	Zeaxanthin	13.296	y = 1.012x + 0.033	0.997	0.062	0.205	95.8–102.4	4.15
3	β-cryptoxanthin	14.325	y = 1.007x − 1.065	0.999	0.025	0.083	92.3–106.9	4.33
4	Canthaxanthin	15.124	y = 1.154x − 1.234	0.994	0.068	0.224	93.5–107.4	4.57
5	Astaxanthin	15.652	y = 1.058x − 0.863	0.992	0.076	0.251	95.8–107.6	4.18
6	Apocarotenal	16.153	y = 1.023x + 0.694	0.991	0.055	0.182	93.1–105.9	3.22
7	Physalien	19.919	y = 1.195x − 0.324	0.995	0.036	0.119	92.8–104.3	2.98
8	β-carotene	22.769	y = 1.102x − 0.753	0.997	0.029	0.096	91.7–109.3	3.74
9	Lycopene	30.032	y = 1.198x − 0.127	0.999	0.041	0.133	96.1–103.2	4.86

^1^ RT—retention time (min), Regression relationships, *R*^2^—coefficient of determination, LOD—limit of detection (ppm), LOQ —limit of quantification (ppm), Recovery (%), CV (%)—coefficient of variation, repeatability.

**Table 3:** Parameters of method validation for tocopherols ^1^.

No.	Tocopherol	RT^1^ (min)	Regression Equation	*R* ^2^	LOD	LOQ	Recovery (%)	CV (%)
1	δ	27.042	y = 1.114x + 0.127	0.998	0.032	0.106	96.4–101.7	2.94
2	γ	83.326	y = 1.076x + 0.247	0.999	0.025	0.083	97.7–103.3	3.23
3	β	104.03	y = 1.012x +0.541	0.997	0.021	0.069	98.5–101.9	3.54
4	α	11.01	y = 1.337x +0.287	0.998	0.026	0.086	97.5–104.2	2.38

^1^ RT—retention time (min), Regression relationships, *R*^2^—coefficient of determination, LOD—limit of detection (ppm), LOQ —limit of quantification (ppm), Recovery (%)-, CV (%)—coefficient of variation, repeatability.

**Table 4:** Statistical parameters for carotenoids ^1^.

μg/g DW	Lut	Zea	Cry	Ast	Apo	Can	Phy	β–Car	Lyc
**Min**	0.046	0.007	0.001	0.005	0.004	0.001	0.004	1.375	0.079
**Max**	0.936	0.109	0.025	0.062	0.024	0.029	0.151	25.800	5.370
**Median**	0.127	0.014	0.007	0.012	0.009	0.004	0.048	7.300	0.384
**St. dev.**	0.136	0.018	0.004	0.010	0.004	0.004	0.031	4.438	0.924

^1^ List of abbreviations is given in Appendix A.

**Table 5:** Statistical parameters for tocopherol.

μg/g DW	δ-Tocopherol	γ-Tocopherol	β-Tocopherol	α-Tocopherol
**Min**	0.001	0.069	0.002	0.022
**Max**	0.010	0.540	0.022	1.331
**Median**	0.003	0.238	0.009	0.528
**St. dev.**	0.002	0.100	0.005	0.238

## Data Availability

All data are presented in this manuscript.

## Supplementary Materials

The following are available online at https://www.mdpi.com/article/10.3390/foods10081956/s1, Table S1: The oil content and fatty acid composition of apple cultivars, Table S2: Composition of saturated fatty acids (SFA), monounsaturated fatty acids (MUFA), polyunsaturated fatty acids (PUFA), Omega-3 fatty acids (Omega 3), Omega-6 fatty acids (Omega 6), Omega-9 fatty acids (Omega 9).

## Appendix A

List of abbreviations. Lut (Lutein), Zea (Zeaxanthin), Cry (β–Cryptoxantine), Ast (Astaxanthin), Apo (Apocarotenal), Can (Canthaxanthin), Phy (Physalien), beta Car (β– Carrotene), Lyc (Lycopene), δT (δ Tocopherol), γT (γ Tocopherol), βT (β Tocopherol), αT (α Tocopherol), Tac (Tocopheryl acetate), Oil (Oil content (%)), Palm (Palmitic acid (C16:0)), Pal (Palmitoleic acid (C16:1(n-7))), Heptdec (Heptadecanoic acid (C17:0)), cis-10-Heptdec (cis-10- Heptadecanoic acid (C17:1)), Stear (Stearic acid (C18:0)), Oleic (Oleic acid), Linoleic (Linoleic acid (C18:2 cis 9,12) n-6), α-Linoleic (α-Linolenic acid (C18:3 cis 9,12,15) n-3), Arach (Arachidic acid (C20:0)), 11-Eicos (11-Eicosenoic acid (20:1 cis-11) n-9), C20, (Eicosadienoic acid (C20:2 cis11,14) n-6), Arachi (Arachidonic acid (C20:4 cis-5,8,11,14) n-6), Heneicos (Heneicosanoic acid (C21:0)), Behenic (Behenic acid (C22:0)), Lignoceric (Lignoceric acid (C24:0)), Docosahex (Docosahexaenoic acid (C22:6 cis 4,7,10,13,16,19) n-3), SFA (saturated fatty acids), MUFA (monounsaturated fatty acids), PUFA (polyunsaturated fatty acids), Omega 3 (Omega-3 fatty acids), Omega 6 (Omega-6 fatty acids), Omega 9 (Omega-9 fatty acids).
