# Supplementary material for: Assessing the Fatty Acid, Carotenoid, and Tocopherol Compositions of Seeds from Apple Cultivars (Malus domestica Borkh.) Grown in Norway
Source: Foods. 2021 Aug 22;10(8):1956. doi: 10.3390/foods10081956 (PMC8392653; doi:10.3390/foods10081956)
Supplement: Supplementary file 1 [file foods-10-01956-s001.zip › Table S1.pdf]

Table S1. The oil content and fatty acid composition of apple cultivars

Cultivar No.	Oil content (%)	Fatty acid composition of oil (%)*															
		Palm	Pal	Heptdec	cis-10-Heptdec	Stear	Oleic	Linoleic	α-Linolenic	Arach	11-Eicos	C20	Arachi	Heneicos	Behenic	Lignoceric	Docosahex
1	18.51	5.93	0.03	0.05	0.03	1.44	28.55	61.12	0.45	1.12	0.44	0.02	0.09	0.05	0.26	0.07	0.34
2	17.11	6.81	<0.01	0.05	0.03	1.53	31.88	57.18	0.28	1.15	0.33	0.08	0.15	0.05	0.19	0.07	0.17
3	15.42	7.47	0.05	0.05	0.03	1.80	31.38	56.07	0.51	1.29	0.40	0.06	0.10	0.06	0.30	0.09	0.25
4	10.10	7.64	0.06	0.04	0.07	1.40	25.42	62.15	0.87	1.03	0.48	0.05	<0.01	0.05	0.27	0.09	0.37
5	7.73	6.99	<0.01	<0.01	<0.01	1.49	21.76	66.73	0.73	0.99	0.36	0.10	0.14	0.08	0.27	0.10	0.16
6	15.98	6.19	<0.01	0.06	<0.01	1.41	30.14	59.01	<0.01	1.17	0.90	0.05	0.11	0.06	0.27	0.11	0.53
7	17.23	7.04	0.04	0.05	0.03	1.46	24.40	64.31	0.50	0.98	0.33	0.08	0.19	0.04	0.25	0.08	0.20
8	12.65	5.75	<0.01	0.07	0.06	1.90	30.49	58.34	0.51	1.48	0.55	0.06	0.12	0.08	0.33	0.10	0.17
9	19.95	5.68	<0.01	0.14	0.10	1.50	28.91	61.14	0.26	1.08	0.31	0.06	0.11	0.04	0.25	0.10	0.37
10	14.54	7.65	0.06	0.05	0.04	1.66	27.61	60.20	0.49	1.09	0.35	0.07	0.12	0.07	0.20	0.10	0.24
11	17.89	6.07	0.03	0.04	0.02	1.59	29.42	60.57	0.28	1.14	0.32	0.04	<0.01	0.03	0.23	0.07	0.14
12	16.27	6.09	0.17	0.03	0.03	1.60	27.20	62.03	0.32	1.35	0.35	0.05	0.13	0.04	0.26	0.08	0.26
13	14.18	7.15	0.05	0.06	0.02	1.33	25.17	63.19	0.57	1.12	0.44	0.08	0.13	0.04	0.36	0.13	0.15
14	19.92	6.82	0.04	0.05	0.05	1.50	32.90	55.66	0.57	1.17	0.51	0.08	<0.01	0.04	0.22	0.16	0.24
15	17.06	6.25	0.10	0.16	<0.01	1.77	35.10	54.20	0.20	1.21	0.27	<0.01	0.13	<0.01	0.27	0.09	0.24
16	16.30	7.13	0.06	0.07	0.05	1.83	28.14	59.67	0.63	1.28	0.36	0.05	0.13	0.02	0.31	0.10	0.17
17	14.47	7.11	0.05	0.07	0.05	1.69	34.82	53.20	0.36	1.36	0.58	<0.01	0.13	0.05	0.30	0.09	0.12
18	14.31	8.47	0.07	0.06	0.03	1.41	27.22	59.79	0.57	1.05	0.39	0.06	0.11	0.05	0.22	0.13	0.36

19	11.58	7.61	0.06	0.05	0.04	1.69	29.59	57.85	0.45	1.23	0.40	0.08	0.12	0.07	0.28	0.09	0.40
20	17.93	6.39	<0.01	0.07	0.03	1.53	29.57	59.51	0.45	1.13	0.44	0.05	0.12	0.05	0.27	0.02	0.30
21	18.13	7.08	0.06	0.10	0.05	1.68	28.17	59.74	0.45	1.20	0.39	0.06	0.09	0.08	0.28	0.09	0.48
22	14.45	7.52	0.05	0.07	0.04	1.53	31.63	55.88	0.53	1.19	0.42	0.04	0.17	0.07	0.25	0.07	0.52
23	9.69	7.68	0.09	0.04	0.05	1.43	31.57	55.71	0.57	1.20	0.44	0.06	0.11	0.10	0.26	0.10	0.60
24	16.73	5.90	<0.01	0.03	0.03	1.42	28.57	61.57	0.32	1.13	0.33	0.05	0.09	0.03	0.23	0.04	0.25
25	19.63	5.90	0.02	0.04	0.02	1.57	31.66	57.92	0.38	1.23	0.47	0.05	0.09	0.04	0.24	0.16	0.27
26	15.69	6.75	<0.01	0.05	0.04	1.53	25.72	62.83	0.53	1.16	0.43	0.07	0.12	0.06	0.26	<0.01	0.35
27	19.98	6.35	0.06	0.06	<0.01	1.66	32.59	55.72	0.40	1.47	0.59	0.07	0.21	0.07	0.32	0.11	0.33
28	22.10	6.28	0.03	0.11	0.02	1.50	29.69	60.09	0.23	1.13	0.33	0.06	0.07	0.04	0.23	0.07	0.11
29	15.34	6.38	<0.01	0.09	0.07	1.45	26.54	62.36	0.49	1.18	0.50	0.05	0.12	0.05	0.26	0.08	0.38
30	12.24	7.43	0.10	0.05	<0.01	1.27	24.82	63.26	0.56	1.16	0.52	0.09	0.10	0.07	0.32	0.10	0.16
31	15.89	6.46	0.07	0.05	0.03	1.80	35.22	53.15	0.52	1.37	0.48	0.05	0.10	0.04	0.32	0.15	0.18
32	9.96	8.55	0.07	0.05	0.03	1.39	24.08	62.45	0.77	1.05	0.41	0.08	0.17	0.07	0.25	0.09	0.49
33	21.21	6.21	<0.01	0.07	<0.01	1.76	26.91	62.48	0.22	1.10	0.57	0.06	0.07	<0.01	0.28	0.09	0.21
34	15.96	6.56	0.04	0.07	0.06	1.62	32.87	55.26	0.52	1.29	0.45	0.04	0.15	0.05	0.28	0.10	0.64
35	13.70	7.04	0.05	0.04	0.06	1.44	26.44	61.88	0.59	1.15	0.45	0.09	0.11	0.05	0.30	0.15	0.15
36	9.71	8.11	0.11	0.08	0.04	1.56	25.52	62.32	0.44	0.96	0.46	0.07	<0.01	0.05	0.24	0.09	<0.01
37	15.42	6.62	0.03	0.07	0.03	1.52	27.31	61.54	0.53	1.17	0.46	0.06	<0.01	0.09	0.24	0.08	0.26
38	19.62	6.11	0.03	0.05	0.04	1.48	31.27	58.29	0.26	1.27	0.36	0.04	0.09	0.05	0.26	0.10	0.30
39	17.64	6.31	0.11	0.06	<0.01	1.78	32.76	56.16	0.47	1.23	0.42	0.05	0.10	0.05	0.30	0.09	0.10
40	16.56	6.85	<0.01	0.09	0.07	1.67	30.04	58.20	0.32	1.25	0.37	0.04	0.10	0.37	0.31	0.14	0.24
41	15.31	6.79	<0.01	<0.01	<0.01	1.28	24.56	64.53	0.52	1.09	0.46	0.11	0.08	0.07	0.30	0.08	0.15

42	8.50	8.27	0.10	0.07	0.05	1.75	28.72	57.79	0.86	1.13	0.33	0.07	0.09	0.05	0.30	0.09	0.27
43	12.84	7.40	0.05	0.06	0.04	1.49	22.97	65.13	0.69	1.03	0.35	0.12	0.14	<0.01	0.27	0.11	0.17
44	18.96	7.10	0.04	0.06	0.03	1.58	34.93	52.66	0.44	1.32	0.57	0.04	0.11	0.04	0.32	0.11	0.64
45	14.73	9.73	0.08	0.08	0.05	2.22	34.77	50.04	0.43	1.30	0.40	0.04	0.10	0.05	0.26	0.12	0.32
46	17.74	6.20	<0.01	<0.01	<0.01	1.72	32.94	56.56	0.20	1.29	0.38	0.06	0.10	0.03	0.22	0.10	0.20
47	9.69	7.84	<0.01	0.04	0.06	1.39	27.13	60.64	0.54	1.12	0.54	0.07	0.11	0.05	0.23	0.11	0.11
48	15.05	7.49	0.07	0.05	0.03	1.84	28.25	59.48	0.48	1.14	0.37	0.05	0.11	0.07	0.29	0.09	0.18
49	19.49	6.12	<0.01	0.09	<0.01	1.58	30.54	58.89	0.42	1.20	0.43	0.07	0.12	0.06	0.28	0.09	0.08
50	12.67	6.66	0.05	0.04	0.03	1.66	27.85	60.21	0.57	1.28	0.50	0.09	<0.01	0.11	0.29	0.09	0.56
51	13.52	6.87	0.06	0.05	0.03	1.34	29.21	59.28	0.44	1.36	0.58	0.07	0.10	0.05	0.29	0.12	0.15
52	8.67	8.09	0.05	0.06	0.04	1.83	33.35	52.95	0.54	1.42	0.49	0.08	0.15	0.06	0.34	0.13	0.43
53	20.94	6.25	0.03	0.03	0.03	1.79	27.47	61.08	0.52	1.28	0.45	0.05	0.13	0.03	0.30	0.10	0.46
54	18.83	6.47	0.08	0.11	<0.01	1.76	24.50	64.39	0.46	1.05	0.37	0.07	<0.01	0.04	0.22	0.17	0.31
55	15.43	6.44	0.04	0.04	0.04	1.50	29.61	59.58	0.42	1.13	0.46	0.05	0.09	0.05	0.22	0.07	0.25
56	18.26	8.90	0.11	0.05	0.04	1.71	31.32	54.05	0.32	1.42	0.54	0.06	0.13	0.07	0.38	0.15	0.74
57	16.34	7.27	0.04	0.05	0.02	1.28	25.78	62.65	0.53	1.11	0.47	0.09	0.10	0.04	0.26	0.08	0.22
58	15.20	7.16	0.04	0.05	0.03	1.51	29.12	58.70	0.53	1.31	0.50	0.06	0.15	0.07	0.36	0.11	0.30
59	19.75	6.71	0.04	0.07	0.03	1.45	30.67	58.69	0.28	1.07	0.33	0.05	0.11	0.07	0.26	0.08	0.16
60	17.75	6.22	<0.01	0.07	0.03	1.71	32.91	56.59	0.23	1.29	0.38	0.07	0.08	0.03	0.22	0.08	0.09
61	19.69	6.90	0.03	0.09	0.04	1.56	27.75	60.38	0.48	1.31	0.45	0.07	0.11	0.04	0.32	0.11	0.33
62	18.87	6.29	0.06	0.05	0.09	1.64	30.36	58.62	0.46	1.21	0.46	0.05	0.13	0.05	0.25	0.08	0.27
63	11.37	7.41	0.06	0.09	0.05	1.71	27.95	59.77	0.49	1.17	0.38	0.06	0.11	0.06	0.33	0.10	0.18
64	20.85	6.43	0.03	0.05	0.11	1.49	27.28	61.94	0.44	1.14	0.43	0.03	0.09	0.09	0.28	0.08	0.15

65	20.22	6.37	0.05	0.06	0.03	1.59	33.21	55.73	0.34	1.21	0.37	0.11	0.15	0.03	0.28	0.12	0.36
66	18.71	6.61	0.05	0.03	0.03	1.41	24.78	63.93	0.53	1.15	0.48	0.08	0.10	0.07	0.28	0.10	0.36
67	14.57	7.06	0.06	0.04	0.02	1.71	27.57	60.45	0.50	1.15	0.31	0.05	0.15	0.06	0.22	0.12	0.51
68	17.18	7.04	<0.01	0.08	0.07	1.53	28.37	60.37	0.26	1.16	0.34	0.05	<0.01	0.06	0.23	<0.01	0.44
69	17.78	5.71	0.07	0.10	<0.01	1.59	32.16	57.78	0.21	1.34	0.36	0.25	0.09	0.08	0.25	0.06	0.25
70	17.59	6.08	0.03	0.03	0.04	1.69	30.09	59.52	0.28	1.19	0.34	0.04	<0.01	0.04	0.29	0.12	0.22
71	17.94	6.16	0.05	0.06	0.04	1.61	26.39	62.84	0.47	1.11	0.42	0.07	<0.01	0.12	0.26	0.10	0.31
72	20.88	5.92	<0.01	<0.01	<0.01	1.84	28.91	60.35	0.37	1.26	0.42	0.10	0.27	0.07	0.30	0.09	0.20
73	18.27	5.85	<0.01	0.06	<0.01	1.65	28.92	60.54	0.41	1.33	0.48	<0.01	0.10	0.06	0.33	<0.01	0.13
74	12.58	7.20	0.07	0.05	0.04	1.56	27.77	60.42	0.51	1.13	0.40	0.06	0.12	0.08	0.28	0.12	0.19
75	15.66	7.11	0.06	0.05	0.03	1.49	29.54	58.18	0.60	1.31	0.56	0.08	0.12	0.07	0.40	0.21	0.20

*Abbreviations correspond to **Palm** (Palmitic acid (C16:0)), **Pal** (Palmitoleic acid (C16:1(n-7))), **Heptdec** (Heptadecanoic acid (C17:0)), **cis-10-Heptdec** (cis-10-Heptadecanoic acid (C17:1)), **Stear** (Stearic acid (C18:0)), **Oleic** (Oleic acid), **Linoleic** (Linoleic acid (C18:2 cis 9,12) n-6), **α-Linoleic** (α-Linolenic acid (C18:3 cis 9,12,15) n-3), **Arach** (Arachidic acid (C20:0)), **11-Eicos** (11-Eicosenoic acid (20:1 cis-11) n-9), **C20**, (Eicosadienoic acid (C20 :2 cis11,14) n-6), **Arachi** (Arachidonic acid (C20:4 cis-5,8,11,14) n-6), **Heneicos** (Heneicosanoic acid (C21:0)), **Behenic** (Behenic acid (C22:0)), **Lignoceric** (Lignoceric acid (C24:0)), **Docosahex** (Docosahexaenoic acid (C22:6 cis 4,7,10,13,16,19) n-3),
