# Supplementary material for: Assessing the Fatty Acid, Carotenoid, and Tocopherol Compositions of Seeds from Apple Cultivars (Malus domestica Borkh.) Grown in Norway
Source: Foods. 2021 Aug 22;10(8):1956. doi: 10.3390/foods10081956 (PMC8392653; doi:10.3390/foods10081956)
Supplement: Supplementary file 1 [file foods-10-01956-s001.zip › Table S2.pdf]

Table S2. Composition of saturated fatty acids (SFA), monounsaturated fatty acids (MUFA), polyunsaturated fatty acids (PUFA), Omega-3 fatty acids (Omega 3), Omega-6 fatty acids (Omega 6), Omega-9 fatty acids (Omega 9)

Cultivar No.	Composition of oil (%)					
	SFA	MUFA	PUFA	Omega-3	Omega-6	Omega-9
1	8.92	29.06	62.02	0.79	61.23	28.99
2	9.85	32.24	57.86	0.45	57.41	32.21
3	11.06	31.86	57.00	0.77	56.23	31.77
4	10.53	26.03	63.45	1.24	62.20	25.90
5	9.90	22.12	67.87	0.90	66.98	22.12
6	9.27	31.04	59.70	0.53	59.17	31.04
7	9.91	24.81	65.28	0.70	64.58	24.74
8	9.72	31.09	59.19	0.68	58.51	31.03
9	8.78	29.32	61.93	0.63	61.30	29.22
10	10.82	28.05	61.13	0.73	60.40	27.96
11	9.18	29.79	61.04	0.43	60.61	29.73
12	9.45	27.76	62.79	0.58	62.21	27.55
13	10.19	25.68	64.12	0.72	63.40	25.61
14	9.96	33.50	56.55	0.81	55.73	33.40
15	9.77	35.48	54.77	0.44	54.33	35.37
16	10.74	28.61	60.64	0.80	59.84	28.51
17	10.68	35.51	53.81	0.48	53.33	35.41
18	11.39	27.72	60.88	0.93	59.96	27.61
19	11.01	30.09	58.90	0.85	58.05	30.00
20	9.46	30.04	60.43	0.75	59.68	30.01

21	10.50	28.67	60.83	0.93	59.90	28.56
22	10.71	32.14	57.15	1.06	56.09	32.05
23	10.79	32.15	57.05	1.18	55.88	32.01
24	8.78	28.93	62.29	0.57	61.72	28.90
25	9.16	32.17	58.70	0.65	58.06	32.12
26	9.81	26.20	63.90	0.89	63.01	26.16
27	10.03	33.23	56.74	0.74	56.00	33.17
28	9.37	30.06	60.57	0.34	60.23	30.01
29	9.49	27.11	63.40	0.87	62.53	27.04
30	10.40	25.44	64.16	0.71	63.45	25.34
31	10.19	35.80	54.00	0.71	53.30	35.70
32	11.45	24.58	63.97	1.26	62.70	24.49
33	9.50	27.48	63.05	0.44	62.61	27.48
34	9.97	33.42	56.61	1.16	55.45	33.32
35	10.18	27.00	62.82	0.74	62.09	26.89
36	11.08	26.12	62.82	0.44	62.39	25.98
37	9.79	27.82	62.39	0.79	61.60	27.76
38	9.33	31.71	58.98	0.56	58.42	31.63
39	9.83	33.30	56.87	0.57	56.30	33.18
40	10.68	30.48	58.90	0.56	58.34	30.41
41	9.60	25.02	65.38	0.67	64.71	25.02
42	11.65	29.20	59.09	1.14	57.95	29.04
43	10.35	23.40	66.24	0.86	65.39	23.31
44	10.54	35.57	53.89	1.09	52.80	35.50
45	13.76	35.31	50.94	0.75	50.19	35.18
46	9.56	33.33	57.11	0.40	56.71	33.33

47	10.79	27.73	61.47	0.65	60.82	27.67
48	10.97	28.73	60.30	0.66	59.64	28.62
49	9.42	30.96	59.57	0.50	59.07	30.96
50	10.14	28.43	61.43	1.13	60.29	28.35
51	10.08	29.88	60.04	0.59	59.45	29.79
52	11.93	33.93	54.14	0.97	53.17	33.85
53	9.79	27.97	62.24	0.98	61.27	27.92
54	9.83	24.95	65.22	0.77	64.46	24.87
55	9.45	30.15	60.40	0.67	59.73	30.07
56	12.68	32.01	55.31	1.06	54.24	31.86
57	10.08	26.32	63.59	0.76	62.84	26.25
58	10.58	29.69	59.73	0.83	58.90	29.62
59	9.70	31.08	59.29	0.44	58.85	31.00
60	9.62	33.32	57.06	0.32	56.74	33.29
61	10.35	28.28	61.38	0.81	60.56	28.20
62	9.57	30.97	59.53	0.73	58.80	30.82
63	10.86	28.44	60.60	0.67	59.94	28.33
64	9.55	27.85	62.66	0.59	62.07	27.71
65	9.66	33.65	56.69	0.70	55.99	33.58
66	9.66	25.33	65.00	0.89	64.11	25.26
67	10.36	27.96	61.68	1.02	60.66	27.88
68	10.10	28.79	61.11	0.70	60.42	28.72
69	9.12	32.59	58.58	0.46	58.12	32.52
70	9.43	30.50	60.06	0.50	59.57	30.43
71	9.42	26.89	63.69	0.78	62.91	26.81
72	9.47	29.33	61.28	0.57	60.71	29.33

73	9.28	29.40	61.18	0.54	60.64	29.40
74	10.42	28.28	61.30	0.70	60.60	28.17
75	10.64	30.19	59.17	0.79	58.37	30.10
